# Protocol for establishing a model for integrated influenza surveillance in Tamil Nadu, India

**DOI:** 10.3389/fpubh.2023.1236690

**Published:** 2023-08-17

**Authors:** Rizwan S. Abdulkader, Varsha Potdar, Gulam Mohd, Joshua Chadwick, Mohan Kumar Raju, S. Devika, Sumit Dutt Bharadwaj, Neeraj Aggarwal, Neetu Vijay, C. Sugumari, T. Sundararajan, V. Vasuki, N. Bharathi Santhose, C. A. Mohammed Razik, Vinoth Madhavan, N. C. Krupa, Nandhini Prabakaran, Manoj V. Murhekar, Nivedita Gupta

**Affiliations:** ^1^National Institute of Epidemiology, Chennai, India; ^2^National Institute of Virology, Pune, India; ^3^Indian Council of Medical Research, New Delhi, India; ^4^Madurai Medical College, Madurai, India; ^5^Government Mohan Kumaramangalam Medical College, Salem, India; ^6^Tiruvarur Medical College Hospital, Tiruvarur, India; ^7^Coimbatore Medical College and Hospital, Coimbatore, India

**Keywords:** influenza, ILI, SARI, model, integrated surveillance, strengthening, capacity building, situation analysis

## Abstract

The potential for influenza viruses to cause public health emergencies is great. The World Health Organisation (WHO) in 2005 concluded that the world was unprepared to respond to an influenza pandemic. Available surveillance guidelines for pandemic influenza lack the specificity that would enable many countries to establish operational surveillance plans. A well-designed epidemiological and virological surveillance is required to strengthen a country’s capacity for seasonal, novel, and pandemic influenza detection and prevention. Here, we describe the protocol to establish a novel mechanism for influenza and SARS-CoV-2 surveillance in the four identified districts of Tamil Nadu, India. This project will be carried out as an implementation research. Each district will identify one medical college and two primary health centres (PHCs) as sentinel sites for collecting severe acute respiratory infections (SARI) and influenza like illness (ILI) related information, respectively. For virological testing, 15 ILI and 10 SARI cases will be sampled and tested for influenza A, influenza B, and SARS-CoV-2 every week. Situation analysis using the WHO situation analysis tool will be done to identify the gaps and needs in the existing surveillance systems. Training for staff involved in disease surveillance will be given periodically. To enhance the reporting of ILI/SARI for sentinel surveillance, trained project staff will collect information from all ILI/SARI patients attending the sentinel sites using pre-tested tools. Using time, place, and person analysis, alerts for abnormal increases in cases will be generated and communicated to health authorities to initiate response activities. Advanced epidemiological analysis will be used to model influenza trends over time. Integrating virological and epidemiological surveillance data with advanced analysis and timely communication can enhance local preparedness for public health emergencies. Good quality surveillance data will facilitate an understanding outbreak severity and disease seasonality. Real-time data will help provide early warning signals for prevention and control of influenza and COVID-19 outbreaks. The implementation strategies found to be effective in this project can be scaled up to other parts of the country for replication and integration.

## Introduction

Acute respiratory infections (ARI) are the most common infectious diseases worldwide and cause significant morbidity and mortality (1). Almost 4 million people die from ARIs every year, of which 98% are due to lower respiratory tract infections (LRI) (2). Influenza viruses cause a significant proportion of these infections (3–5). The economic loss due to respiratory infections caused by influenza viruses was estimated to be between US$71 and US$167 billion annually (6). The potential of influenza viruses to cause public health emergencies in society, evidenced by several incidents in the past, such as the Spanish flu of 1918, the Asian influenza of 1957, the Hong Kong influenza of 1968 and the H1N1 pandemic of 2009, cannot be overstated (7–10).

International committees convened by the World Health Organization (WHO) had found that the world was unprepared to respond to an influenza pandemic. The International Health Regulations 2005 require that each member state develop and maintain capabilities to detect, assess, and report disease events nationally and internationally to the WHO within 48 h of confirmation (11). However, reviews of national pandemic planning indicate that surveillance systems are often inadequate to support current preparedness strategies (12–16), especially in low-and middle-income countries (LMIC) like India and the available surveillance guidelines for pandemic influenza lack the specificity that would enable many countries to establish operational surveillance plans (17, 18). Also, the WHO has proposed a global influenza strategy 2019–2030, which focuses on improving global research and innovation to fill the gaps in our current knowledge about the transmission and host response of the influenza viruses, strengthen the influenza surveillance and pandemic preparedness and to expand the seasonal influenza prevention and control policies (19).

A routine sentinel surveillance system for influenza will gather data that can aid healthcare policy makers and providers in making decisions regarding program management and patient care (18). In India, influenza-like illness (ILI) and severe acute respiratory infection (SARI) surveillance are only partially implemented as part of the National Integrated Disease Surveillance Programme (IDSP). There are several pitfalls in the existing surveillance system. Lack of information sharing between surveillance reporting units and feedback from higher centres, combined with poor reporting quality and lack of epidemiological information crucial to identify and responding to outbreaks, result in poor disease surveillance and pandemic preparedness. Moreover, information on the current knowledge, practice and problems associated with the existing influenza surveillance system is scarce and it should be assessed to address problems and formulate solutions in order to get quality data for enhanced public health response (20, 21).

Recently, a strong need to include more epidemiological information to complement the virological data (18) for better understanding the influenza epidemiology and it severity has been emphasized globally (22–24). In the long run, establishment of a model for influenza and related infections surveillance will guide national approaches to optimal vaccination policies and appropriate allocation of healthcare resources (25). Previous studies have highlighted the need for a strong surveillance system to enhance and strengthen a country’s capacity to detect and prevent seasonal, novel, and pandemic influenza (17).

There are several variations of influenza surveillance systems across the developed world. Most of these nations follow the WHO guidance for influenza surveillance (virological surveillance, primary care surveillance and hospital surveillance) (18). For example, the European influenza surveillance network (EISN) by European Centre for Disease Prevention and Control (ECDC) is responsible for the combined epidemiological and virological surveillance for influenza in European Union countries. Moreover these systems are complemented by initiatives like Influenzanet and EuroMOMO project for community surveillance and mortality surveillance, respectively (26, 27). The U.S. WHO Collaborating Laboratories System and National Respiratory and Enteric Virus Surveillance System (NREVSS) are responsible for the virological surveillance in US whereas the outpatient illness and hospital surveillance are monitored by the ILINet and FluSurv-NET, respectively (28). In developing countries, on the other hand, there are very few examples of such organized systems of surveillance for influenza (29–31).

Globally, the number of countries conducting routine influenza surveillance has increased over the last decade (32). Lessons from existing surveillance systems suggest that countries need to set up a robust surveillance system with components in both hospital and community settings and include epidemiologic and virologic aspects of data collection. Another important recommendation is to use data for action, especially as a tool for early detection and response to outbreaks (27, 28, 33, 34). However, it is also true that setting up developed countries’ style systems can be very resource intensive and impractical for low-and middle-income countries like India. For example, countries like China and Malaysia have tried to set up surveillance systems but lack some components of influenza surveillance, such as sentinel surveillance in primary care, non-medically attended surveillance, mortality surveillance and integration with the routine health system (31).

Keeping these lessons in mind, we have incorporated some lessons from the ongoing global surveillance programs. In our proposed surveillance, we will be incorporating joint virologic and epidemiologic surveillance at primary care and sentinel hospital levels. This will also be integrated into the existing routine health systems to ensure sustainability. In order to ensure this integration, staff of the routine health care system will be regularly trained to carry out the surveillance activities and perform advanced data analysis for informing policy and action. Another important addition to the project is the mortality and disability surveillance which will be performed for both ILI and SARI cases. Since routine health systems are already burdened with several other priorities, we will integrate surveillance for influenza with other major pathogens such as SARS-CoV-2. Finally, we will be selecting sentinel sites which represent different climatic and geographical conditions.

Tamil Nadu is the 11th largest state situated in the southern part of India. The State is divided into 32 districts and estimated to have a population of over 7.21 crores which accounts for approximately 5.94% of India’s total population (35). According to National Centre for Disease Control—India, Tamil Nadu state reported 2,827 cases and 25 deaths associated with influenza A (H1N1) compared to 13,202 cases and 410 deaths in the entire country during 2022 (36). However, these figures are likely to be gross underestimates of the actual burden since there is no on-going robust surveillance system for influenza in India. In order to address these circumstances, a well-designed epidemiological and virological surveillance is required to understand local influenza epidemiology, detect and respond to outbreaks, establish disease burden, detect mutants and identify emerging new strains. In this paper, we present a study protocol for establishing a novel integrated influenza surveillance model in Tamil Nadu, India.

## Study objectives

**Figure 1:**
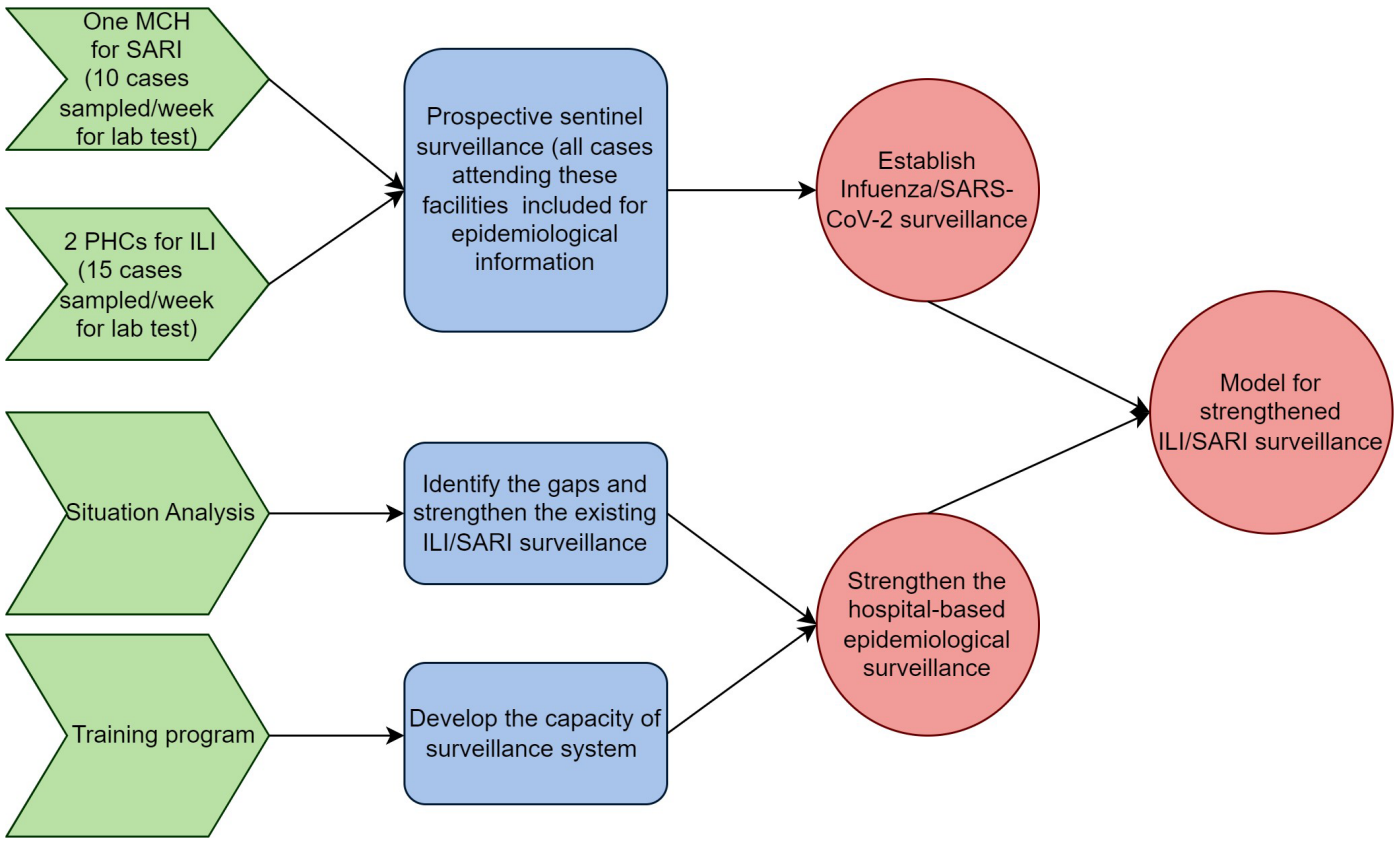
Schematic representation of objectives and activities of the model for integrated influenza surveillance in Tamil Nadu, India (MIST) project.

This project aims to develop a model of strengthened ILI/SARI surveillance for the country through the following objectives (Figure 1).

Objective 1: to establish influenza and SARS-CoV-2 surveillance in four identified districts of Tamil Nadu, India.

Objective 2: to strengthen the hospital-based epidemiological ILI/SARI surveillance in the identified districts of Tamil Nadu with the following sub-objectives

- Identify the gaps and strengthen the existing ILI/SARI surveillance.
- Develop the capacity of the surveillance system for undertaking advanced epidemiological analysis of surveillance data.

## Materials and methods

**Figure 2:**
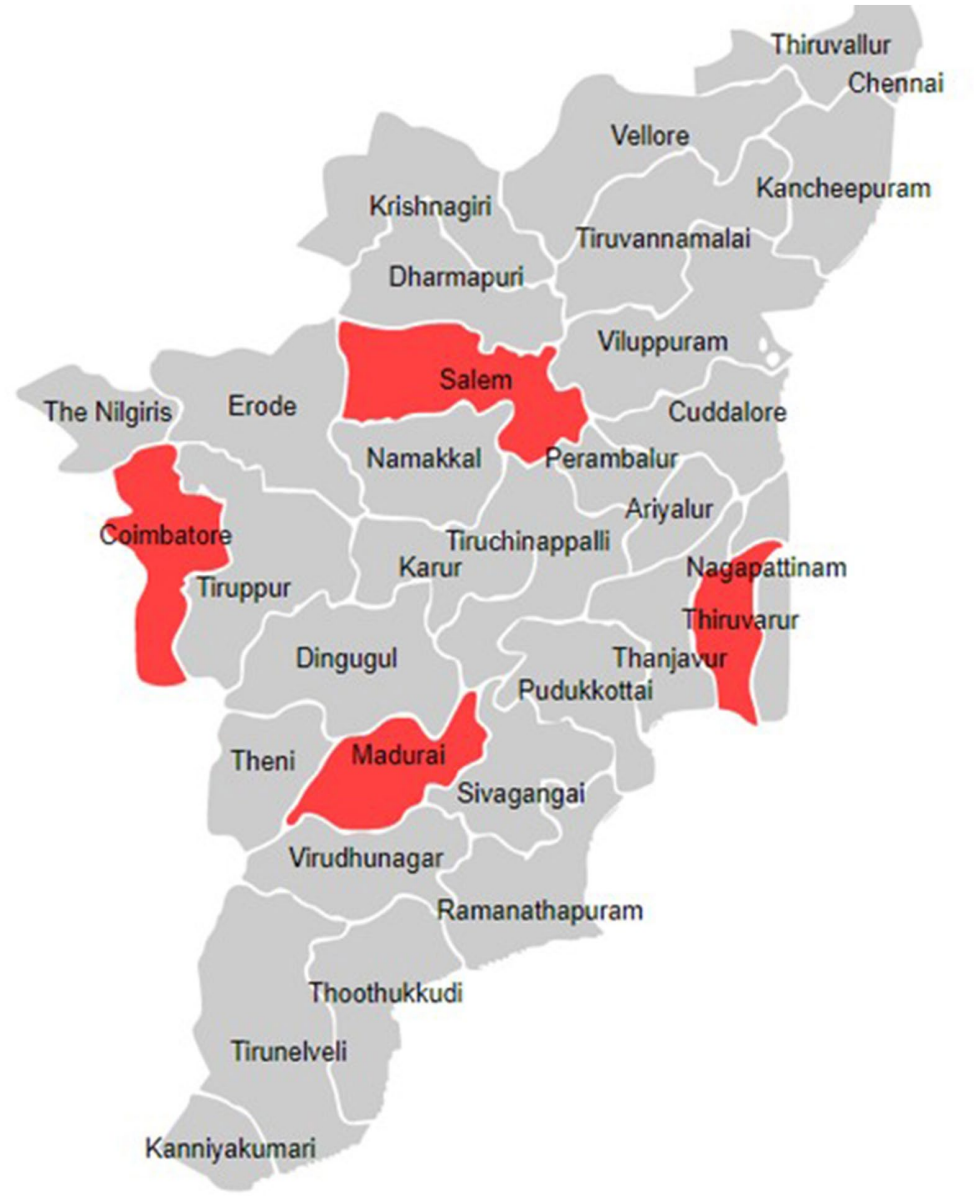
Location of the districts included in the model for integrated influenza surveillance in Tamil Nadu, India (MIST) project.

This project will be carried out as implementation research in four selected districts of Tamil Nadu (Figure 2). One medical college hospital and two primary health care centres (PHCs) will be selected in each district to establish prospective sentinel surveillance, from which the SARI and ILI-related information will be collected, respectively. We chose primary care centres as sentinel sites for mild to moderate infections which will be captured under the influenza-like illness (ILI) definition and medical colleges as sentinel sites for severe infections which will fall under the severe acute respiratory infections (SARI) case definition. The number of districts and health care facilities selected for this study was based on available financial resources and the need for optimal geographical representation. The intended time period for completing this project will be 3 years.

### Methods for objective 1

#### Study design

We will adopt a prospective sentinel surveillance approach.

#### Study setting

We will select one medical college hospital and two PHCs in each district to collect information related to SARI and ILI, respectively.

#### Study population

We will include all ILI/SARI patients of all age groups attending the selected healthcare facilities.

#### Study definitions

A case with an acute respiratory infection accompanied by a fever of ≥38°C and cough with onset within the last ten days will be considered as ILI. A case with an acute respiratory infection accompanied by a fever of ≥38°C, and cough with onset within the last ten days requiring hospitalization will be considered SARI.

#### Samples size

All patients attending the identified healthcare facilities will be included for epidemiological surveillance. The number of nasopharyngeal samples to be tested was determined based on several factors, including the available testing budget, the capacity of the laboratory, manpower available and the population size under surveillance. We have decided to collect 10 SARI and 15 ILI samples per site per week. This will lead to a collection of 1,300 samples per year for three years (total of 5,200 samples). This sample size was considered appropriate to determine the current strains of viruses circulating in the community in a given week. The total budget available for testing samples will be equally allocated to the four project sites. The primary purpose of virological testing was to inform the public health decision makers of the nature of the outbreak, if any, and the emergence of any novel or unknown strains and not as a part of clinical diagnostic requirement.

#### Study tool

A pre-tested and structured case reporting form (CRF) will be utilized to collect information from ILI and SARI cases. The CRF will collect personal characteristics, comorbidity status, current illness, factors contributing to the illness, clinical features, treatment given, and outcomes (Supplementary material).

#### Laboratory procedures for virological testing

**Figure 3:**
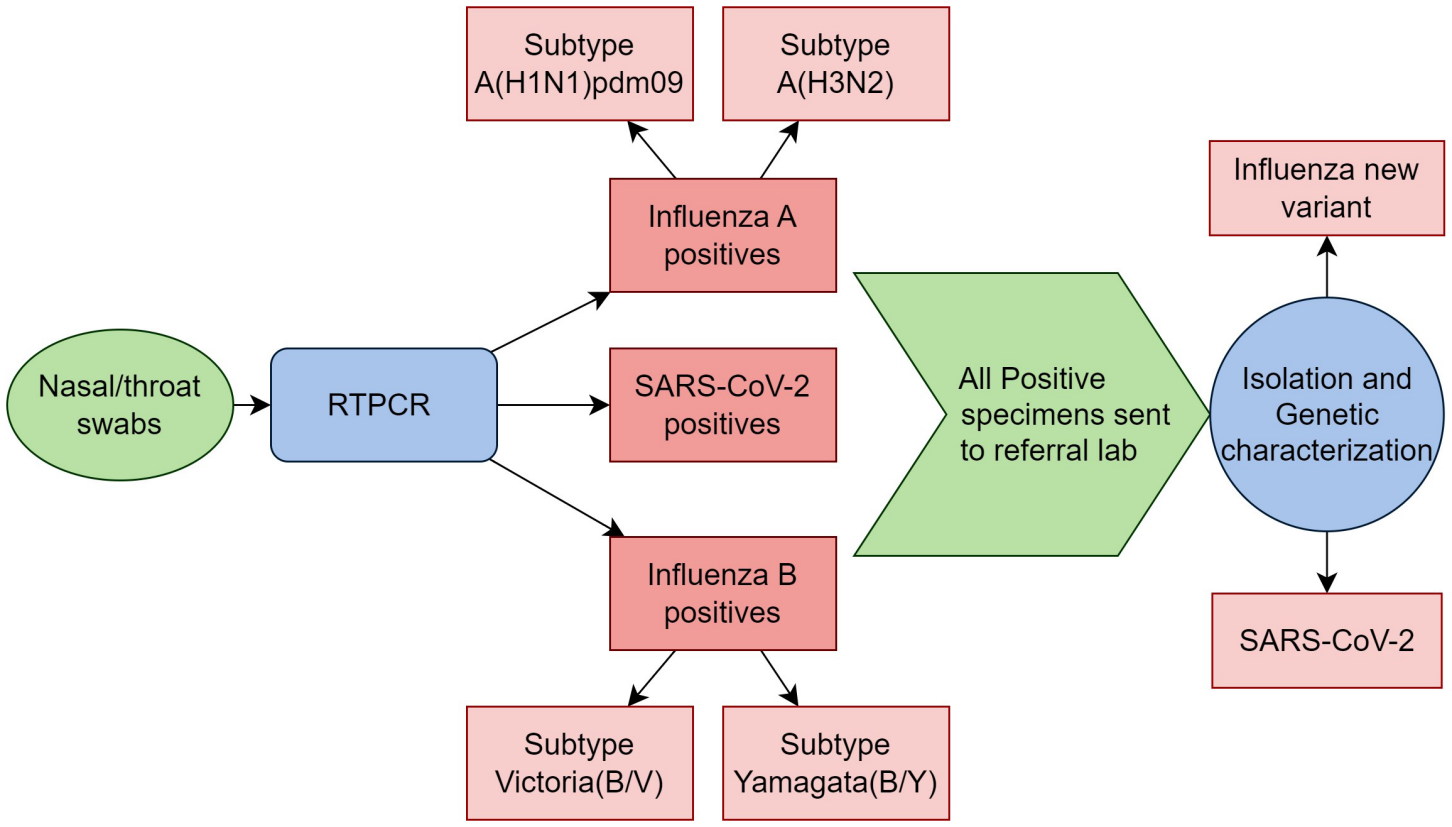
Schematic representation of laboratory procedures in the model for integrated influenza surveillance in Tamil Nadu, India (MIST) project.

Clinical specimens, including nasal, throat, and combined nasal/throat swabs, will be collected and preserved in 2–3 mL of viral transport medium (VTM), stored at 4°C, and transported to the laboratory within 4 h. Real-time polymerase chain reaction (RT-PCR) method will be used for molecular analysis of the samples (SARS-CoV-2 and influenza) using a multiplex assay kit containing three primer/probe sets (influenza A, influenza B, SARS-CoV-2) targeting the RNA of influenza A, influenza B, and SARS-CoV-2. The primers/probes will detect influenza A viruses from a conserved region of the matrix (M1) gene and influenza B virus from the non-structural gene (NS2). The influenza A positive samples will further be subtyped for A (H3N2) and A (H1N1)pdm09, and influenza B positive samples for Yamagata and Victoria (B/Y, B/V). Positive clinical specimens will be shipped to the referral lab earmarked by the sentinel lab for isolation and genetic characterization for SARS-CoV-2 and influenza novel variants. All laboratory procedures will be performed following standard operating procedures (Figure 3).

#### Data collection and analysis plan

Trained project staff will collect samples for testing and epidemiological information. Separate CRFs (Annexure 1) will be used for ILI and SARI-related data collection which includes demographic, clinical, hospitalization, management & outcome details. The collected data will be entered into the REDCap platform and analysed weekly for quality and completeness. District and pathogen-wise data analyses will be carried out weekly and reported to the appropriate authorities. A real-time dashboard will be developed, maintained, and shared with the authorities for displaying the total number of SARI/ILI cases enrolled and trends in test positivity for influenza viruses A & B and SARS-CoV-2 by week, age group and district. Analysed data will be expressed as frequency/proportion in the case of categorical variables and as the mean and standard deviation in the case of continuous variables.

#### Quality assurance

The project site laboratories will undergo periodic inspections to ensure testing quality. An external quality assurance system will be established through the laboratory at the National Institute of Virology, Pune. A periodic check for the quality of the data entered will be carried out, and daily and weekly review meetings will be conducted for the project staff to ensure proper data collection and reporting.

### Methods for objective 2

#### Objective 2.1. Situation analysis of surveillance systems

The surveillance for ILI and SARI is a critical component of the public health response to these diseases. However, implementing ILI/SARI surveillance has many challenges, such as inadequate resources, lack of standardization, and insufficient manpower and training. To identify these gaps and challenges, we will conduct a situation analysis of the ILI/SARI surveillance system in the study districts.

#### Study design

We will adopt a mixed-methods approach.

#### Study setting

We will conduct the study in the selected health facilities of the study districts.

#### Study population

All personnel involved in ILI/SARI surveillance in the selected facilities will be included in the study.

#### Sampling and sample size

One medical college each from the government and private sectors, one district hospital, two sub-district hospitals, a sample of PHCs, two private hospitals and one microbiology laboratory (one each from the government and private sectors) will be selected by convenience. In addition to these, we will interview the personnel of the district and state surveillance units.

#### Study tools

We will use interview guides for carrying out in-depth interviews with study participants and observation check-lists for health facilities based on the components of the WHO situation analysis tool (37) (Supplementary material).

#### Data collection and analysis plan

We will describe the current status of the surveillance system in terms of the availability of human resources and facilities, data collection process, analysis of samples, reporting methods and the training requirements from the facility checklist and in-depth interviews. We will interview the nodal person involved in the ILI/SARI surveillance at different levels, collect information regarding their views and challenges on ILI/SARI surveillance, and obtain their recommendations to improve it. Quantitative variables will be expressed in mean and SD and frequencies and percentages. Qualitative data will be transcribed into English, and a thematic analysis will be carried out.

#### Strategies for strengthening the surveillance system

Based on the situation analysis, we will identify the existing gaps in the surveillance system. We will advocate for strengthening identified laboratories by optimizing logistics supply, training testing staff regularly, and implementing an enhanced data management system. These measures will be implemented in the identified laboratories within the project districts, bolstering their overall capacity.

We will develop and share with the district health authorities the surveillance manuals, standard operating procedures (SOPs) for data collection, sample collection, sample transport, data collection tools, and data analysis format. Nodal persons will be identified in the concerned departments to ensure sustained collaboration and undertake required initiatives whenever required. SOPs will also be developed for information exchange and regular meetings of stakeholders to ensure sustained collaboration between the concerned departments. Periodic reports will be sent to district and state officials to coordinate response activities to ensure the effectiveness of the ILI/SARI surveillance.

### Objective 2.2. Develop the capacity of the surveillance system for analysis of surveillance data

To enhance the effectiveness of the ILI/SARI surveillance system, we need to develop the capacity within the system for routine analysis of surveillance data. To achieve this, we will train the district and state-level staff involved in ILI/SARI surveillance for epidemiological data collection, collation, analysis, and reporting. We will utilise the situation analysis results to develop training sessions targeting the knowledge gap within the disease surveillance system. These training sessions will be designed to address and bridge any existing gaps in their understanding and expertise. The training program will be conducted in batches of 20 members at state headquarters until all personnel are trained.

By providing this training, we aim to empower the staff to efficiently and effectively analyse surveillance data, enabling the concerned authorities to make data-driven decisions and take proactive measures to control the spread of infectious diseases.

### Ethics statement

The study protocol has been approved by the Institutional Human Ethics Committee (IHEC) of all the participating sites. The study participants will be given details of the study objectives and data collection methods. Age-appropriate written informed consent will be obtained. Complete confidentiality and anonymity of the identifiers and information collected will be maintained. Personal identifiers will be available only in the data collection proforma and not used during the analysis or publication/dissemination. Biological samples such as nasal/nasopharyngeal swabs will be collected and tested as per standard laboratory procedures. During follow-up, if any participant requires medical attention, they will be referred to the appropriate hospital for further management.

## Discussion

Implementing an integrated approach for collecting virological and epidemiological surveillance data, combined with the potential of advanced analysis, could strengthen the country’s efforts for preparedness during public health emergencies (28, 38, 39). Good quality historical surveillance data can be used to understand better and answer critical questions such as the severity and seasonality of outbreaks and help compare trends between various regions of the country and even between different countries (40).

India has faced several epidemics and pandemics in the past (7). However, because of the lack of communication and coordination in surveillance, it is practically impossible to predict and prepare for any pandemic occurring in the future. Laboratory detection for the viruses is the only way to confirm the diagnosis of a particular respiratory viral infection, as all respiratory viral infections have overlapping symptoms (41). This project will use the existing Virus Research Diagnostic Laboratory (VRDL) network labs attached to the study site for the lab confirmation of influenza and SARS-CoV-2. This will strengthen the existing surveillance and help administer timely antiviral treatment to patients to reduce the duration of symptoms and prevent transmission to others (3). Also, epidemiological data collection of ILI/SARI cases will help provide specific disease transmission and impact indicators. This surveillance model and its outputs will provide the health system with valuable tools for conducting advanced analysis of surveillance data and detect warning signals of potential outbreaks in a timely manner. These capabilities greatly enhance the development of prevention and control policies, including the implementation of effective vaccination strategies and specific non-pharmacological interventions such as mask mandates, quarantine and isolation measures. By leveraging the insights derived from this model, the health system will be empowered to make more informed decisions and optimize their approaches for mitigating the spread of influenza and other respiratory viruses.

The strategies adopted in this project have several strengths. First, we base our interventions on the situation analysis of the existing ILI/SARI surveillance system to identify the needs and gaps. Based on these findings we will develop “plug-ins” in the existing surveillance system to further strengthen it. Second, real-time data analysis and presenting it in the form of an easily accessible dashboard will help provide early warning signals and guide strategies for prevention and control. Lastly, we will train the staff involved in disease surveillance with standard definitions for ILI/SARI for correctly identifying and reporting ILI/SARI cases from the community as part of situation analysis and training.

There are a few limitations in this implementation project. We intend to test only for influenza A, influenza B and SARS-CoV-2. Other dominant respiratory viruses like respiratory syncytial virus (RSV) which may cause severe disease among high-risk groups (HRGs) are not included in the testing algorithm (42, 43). Since the surveillance method used here is of the sentinel type, we will miss cases occurring outside the catchment areas of the sentinel sites (44).

## Ethics statement

The studies involving humans were approved by Institutional Human Ethics Committee, ICMR—National Institute of Epidemiology. The studies were conducted in accordance with the local legislation and institutional requirements. Written informed consent for participation in this study was provided by the participants’ legal guardians/next of kin.

## Author contributions

RA, VP, NG, GM, JC, MR, SD, MM, SB, NA, and NV were responsible for conceptualization, design, and development of the methodology. RA, CM, and VM drafted the initial manuscript. VP, NG, GM, JC, MR, SD, MM, SB, NA, NV, CS, TS, VV, NB, NK, and NP provided the critical comments to the manuscript. All authors contributed to the article and approved the submitted version.

## Funding

This work was funded and supported by the Indian Council Medical Research (Project grant ID-R.15013/10/2021-HR-VRDL). The funding body had no role in the design of the study and collection, analysis, and interpretation of data and in writing the manuscript.

## Conflict of interest

The authors declare that the research was conducted in the absence of any commercial or financial relationships that could be construed as a potential conflict of interest.

## Publisher’s note

All claims expressed in this article are solely those of the authors and do not necessarily represent those of their affiliated organizations, or those of the publisher, the editors and the reviewers. Any product that may be evaluated in this article, or claim that may be made by its manufacturer, is not guaranteed or endorsed by the publisher.
