# Supplementary material for: Protocol for establishing a model for integrated influenza surveillance in Tamil Nadu, India
Source: Front Public Health. 2023 Aug 17;11:1236690. doi: 10.3389/fpubh.2023.1236690 (PMC10469860; doi:10.3389/fpubh.2023.1236690)
Supplement: Supplementary file 1 [file Data_Sheet_1.PDF]

Supplementary materials

Protocol for establishing a model for integrated Influenza surveillance in Tamil Nadu, India.

Authors:

Rizwan S Abdulkader*[1], Varsha Potdar[2], Gulam Mohd[1], Joshua Chadwick[1], Mohan Kumar Raju[1], Devika S[1], Sumit Dutt Bharadwaj[2], Neeraj Aggarwal[3], Neetu Vijay[3], Sugumari C[4], Sundararajan T[5], Vasuki V[6], Bharathi Santhose N[7], Mohammed Razik C A[1], Vinoth Madhavan[1], Krupa N C[1], Nandhini Prabakaran[1], Manoj V Murhekar[1], Nivedita Gupta[3].

Author's affiliation:

[1]National Institute of Epidemiology, Chennai, India.

[2]National Institute of Virology, Pune, India

[3]Indian Council of Medical Research, New Delhi, India

[4]Madurai Medical College, Madurai, India

[5]Government Mohan Kumaramangalam Medical College, Salem, India

[6]Tiruvarur Medical College Hospital, Tiruvarur, India

[7]Coimbatore Medical College and Hospital, Coimbatore, India

***Corresponding Author:**

Dr. Rizwan S Abdulkader,
Scientist – D,
R-127, TNHB Colony, ICMR-NIE, Chennai – 77.
Email: sarizwan1986@nieicmr.org.in,
Mobile no: +91 8447284098

Annexure 1

CRF: Patients with Severe Acute Respiratory Illness (SARI)

**CRF ID:**									

**I. Screening Questions**	
1.1. Whether the patient has been interviewed for the same before?	1. Yes 2. No
1.2. Does the patient satisfy SARI definition (an acute respiratory infection with measured fever >=38ºC or >=100.4ºF and cough with onset in the last 10 days)	1. Yes 2. No
1.3. Consent given: 1. Yes 2. No	1.4. Decision: 1. Include 2. Exclude

**II. Socio-demographic information**	
2.1. Hospital IP no:	2.6. Home address:
2.2. Name:	
2.3. Date of enrollment: dd/mm/yyyy	2.7. Pin code:
2.4. Age (in completed years):	2.8. Primary contact no:
2.5. Gender : 1. Male 2. Female 3. Transgender	2.9. Secondary contact no:

2.10. Nature of locality	1. Rural 2. Urban	3. Slum	Urban slum
			Rural slum

2.11. Profession	1. Farmer<br>2. Businessman<br>3. Clerical work	4. Student<br>5. Teacher<br>6. Livestock farming<br>7. Trading	8. Poultry farming<br>9. Health care worker<br>10. Pig farmer<br>11. Butcherer	12. Others, specify ______________

2.12. Type of home	1. Concrete standalone home<br>2. Housing society	3. Semi pukka<br>4. Kaccha

2.13. No. of persons living in the house_______________	2.14. No. of children(<18 years) living in the house ____________

2.15. Cooking fuel used in the house	1. LPG<br>2. Firewood	3. Cow dung<br>4. Kerosene oil	5. Others______________

2.16.Separate place(Kitchen) for cooking in the house	1. Yes 2. No
2.17. Do you often undertake intercity/long-distance travel because of your job/family? **<u>(routine travel)</u>**	1. Yes 2. No
2.17.1. If yes, the mode of travel used?	1. Personal vehicle 2. Train 3. Bus 4. Air 5. Others___________________
2.18.1. Influenza vaccination within last one year	1. Yes 2. No
2.18.2. COVID-19 Vaccination	1. Yes 2. No

If 'yes' type of vaccine received? 1.Covaxin 2. Covishield 3. Sputnik V, 4. Others, specify ___________________ 5. Not Known

		If 'yes'-Name	If 'yes'-Date
2.18.2.1. 1st dose	1.Yes 2. No	.	dd/mm/yyyy
2.18.2.2. 2nd dose	1.Yes 2. No		dd/mm/yyyy
2.18.2.3. Booster	1.Yes 2. No		dd/mm/yyyy

2.19. Routine immunization status (Under five child)	1.Fully immunized 2. Partially immunized 3. Unimmunized
2.20. Treatment taken in the last two weeks : 1. Antivirals 2. Antibiotics 3. Not taken	

**III. Patient Symptom details**	
3.1. First symptom (multiple)	1.Fever 2. Sore throat 3. Cough 4. Cold 5. Diarrhea 6. Others_____________
3.2 Date of onset of first symptom: dd/mm/yyyy	3.3 Date of first health care seeking: dd/mm/yyyy
3.4 First health facility visited: 1. Private clinic/ hospital 2. Government hospital 3. Others____________________________	
3.5 Type of treatment availed in the first facility	1. OPD 2. IPD care__________________ days
3.6 Whether referred to the current facility	1. Yes, date of referring <u>dd/mm/yyyy</u> 2. No
3.7 Date of admission in the current facility	dd/mm/yyyy

3.13. Earache			3.26. Nausea/ Vomiting		
3.14. Wheezing			3.27. Diarrhea		
3.15. Chest pain			3.28. Conjunctivitis		
3.16. Myalgia			3.29. Skin rash		
3.17. Arthralgia			3.30. Skin ulcers		
3.18. Fatigue/ Malaise			3.31. Lymphadenopathy		
3.19. Shortness of breath			3.32. Loss of smell		
3.20. Lower chest wall in drawing (under five)			3.33. Loss of taste		
3.34. Other symptoms: 1. Yes, specify____ 2. No					
**IV. Pre-existing Medical condition**	**Yes**	**No**	**IV. Pre-existing Medical condition**	**Yes**	**No**
4.1. Cardiac diseases			4.10 Malignancy		
4.2. Hypertension			4.11 Neurologic diseases (dementia)		
4.3. Diabetes Mellitus			4.12 Immunodeficiency disorders		
4.4. Chronic Kidney diseases			4.13 Anaemia		
4.5. Asthma			4.14 Hemoglobinopathy		
4.6 COPD/Emphysema			4.15 HIV infection		
4.7 Tuberculosis			4.16 Rheumatologic disease		
4.8 Other chronic lung diseases			4.17 Recurrent fever prior to admission		
4.9 Hepatic diseases			4.18 Any other chronic illness (specify if 'yes')____________		

**V. Risk Factors**		
5.1. Receiving immunosuppressive medications, including inhaled/oral steroids		1.Yes, specify ___________________2. No
5.2. Tobacco use	Smoke	1.Daily smoker 2. Occasional smoker 3. Ex-Smoker 4. Never smoker
	Smokeless	1.Daily use 2.Occassional use 3. Previously used 4. Never used
5.3. Alcohol use		1.Daily drinker 2. Occasional drinker 3. Ex-drinker 4. Never drinker
5.4. Injectable drug use		1. Yes 2. No
5.5. Current pregnancy		1.Yes, gestational week(s)________2. No
5.6. Post-partum (≤6 weeks)		1. Yes 2. No
5.7. H/O travel in the 14 days prior to the first symptom onset		1. Yes 2. No
5.8. If Q.5.6 is 'Yes' , specify the location		1.Within the state 2. Outside the state 3. Outside the country
5.9. Any pets in the home		1.Yes, specify _______________ 2. No
5.10. Exposure to birds / poultry/dead bird in last 14 days		1.Yes, specify _______________ 2. No
5.11. Exposure to farm/live animal market/zoo/wild animals in the past 14 days		1.Yes, specify _______________ 2. No
5.12. H/O attending any type of gatherings (wedding, parties, religious meetings etc. other than your workplace) in the past two weeks		1.Yes, specify _______________ 2. No
5.13. In the past 14 days prior to symptoms onset, did you have any contact with a family member/relative/anyone in the neighbourhood or workplace having the similar illness		1.Yes, Contact____________________ 2. No
5.14. H/o previous hospital admission for a similar illness in the past one year		1. Yes 2. No
5.14.1. If yes, whether admitted in ICU/HDU, the total duration		_________Days
5.15. H/O lab confirmed Covid-19 positive in past one year		1. Yes 2. No
5.16. If covid positive, treatment availed		1.Home 2. In patient care

**VI. Clinical signs at admission (Record the first value in the first 24 hours of admission for each vital sign)***	
6.1. Weight (kg)	
6.2. Height (cm)	
6.3. Mid-Upper arm circumference (Under five) (cm)	
6.4. Axillary temperature (Degree Celsius)	
6.5. Heart rate (beats per minute)	
6.6. Respiratory rate (per minute)	
6.7. Blood pressure (mm of Hg)	SBP____________ DBP___________
6.8. Severe dehydration	1.Yes 2. No
6.9. Urine flow rate (urine output chart)	_______(in ml/24 hours)
6.10. Wheeze	1. Yes 2. No
6.11. Stridor in calm patient	1. Yes 2. No
6.12. Crepitations on auscultation	1. Yes 2. No

6.13. Oxygen saturation at room air (%)	
6.14. Oxygen saturation with supplemental O2 (%)	
6.15. Glasgow coma scale (The lowest value between 3-15 or NA, if not documented)	
6.16. Sternal capillary refill time >2secs (Under five)	1. Yes 2. No
6.17. Nasal flaring (Under five)	1. Yes 2. No
6.18. Grunting (Under five)	1. Yes 2. No
**VII. Complications** ***(at any time during hospitalization)****	
7.1. Viral pneumonitis	1. Yes 2. No
7.2. Bacterial pneumonia	1. Yes 2. No
7.3. Acute lung injury / ARDS	1. Yes 2. No
7.4. Pneumothorax	1. Yes 2. No
7.5. Pleural effusion	1. Yes 2. No
7.6. Bronchiolitis	1. Yes 2. No
7.7. Meningitis/Encephalitis	1. Yes 2. No
7.8. Seizures	1. Yes 2. No
7.9. Stroke	1. Yes 2. No
7.10. Congestive heart failure	1. Yes 2. No
7.11. Endo/myo/peri-carditis	1. Yes 2. No
7.12. Cardiac arrhythmia	1. Yes 2. No
7.13. Cardiac ischemia	1. Yes 2. No
7.14. Cardiac arrest	1. Yes 2. No
7.15. Bacteraemia	1. Yes 2. No
7.16. Coagulopathy or DIC	1. Yes 2. No
7.17. Anaemia	1. Yes 2. No
7.18. Rhabdomyolysis or myositis	1. Yes 2. No
7.18. Acute renal injury/failure	1. Yes 2. No
7.20. Gastrointestinal bleeding	1. Yes 2. No
7.21. Pancreatitis	1. Yes 2. No
7.22. Hepatic dysfunction	1. Yes 2. No
7.23. Hyperglycaemia	1. Yes 2. No
7.24. Hypoglycaemia	1. Yes 2. No
7.25. Other complication	1.Yes , specify__________________ 2. No
7.26.Urinary tract infection	1. Yes 2. No

**VIII. Treatment details** ***(at any time during hospitalization)****		
8.1. Supplemental oxygen via mask or nasal cannula, or hood	1. Yes 2. No	
	Starting date: dd/mm/yyyy	Weaning date: dd/mm/yyyy
8.2. Mechanical ventilation	1. Yes 2. No	
	Starting date: dd/mm/yyyy	Weaning date: dd/mm/yyyy
8.3. CPAP / BiPAP	1. Yes 2. No	
	Starting date: dd/mm/yyyy	Weaning date: dd/mm/yyyy
8.4. Renal replacement therapy (RRT)/ Dialysis	1. Yes 2. No	
8.5. Inotropes/ Vasopressors	1. Yes 2. No	
8.6. Plasmapheresis/ Exchange transfusion	1. Yes 2. No	
8.7. Intravenous immunoglobulin	1. Yes 2. No	
8.8. Oral rehydration therapy / IV fluids	1. Yes 2. No	
8.9. Blood transfusion or products	1. Yes 2. No	

**8.10 Drugs given (Multiple)***				
**Antivirals**		**Antibiotics**		**Antifungals**
1.Oseltamivir<br>2.Baloxavir<br>3.Zanamivir<br>4.Peramivir<br>5.Remdesivir	6.Paxlovid<br>7.Molnupiravir<br>8.MAB cocktail<br>9.Any other Antiviral<br>_____________	1.Azithromycin<br>2.Doxycycline<br>3.Moxifloxacin<br>4.Vancomycin<br>5.Ceftriaxone<br>6.Levofloxacin	7.Amoxiclav<br>8.Clarithromycin<br>9.Piperacillin Tazobactam<br>10.Cefaperazone Sulbactam<br>11.Any other Antibiotic<br>______________________	1.Voriconazole<br>2.Amphotericin B<br>3.Fluconazole<br>4.Any other Antifungals<br>_________________

**8.11 Corticosteroids**	**8.12 Anticoagulants**	**8.13 Bronchodilators**

1.Betamethasone<br>2.Dexamethasone<br>3.Hydrocortisone<br>4.Methyl prednisolone	5. Prednisolone<br>6.Fluticasone<br>7.Beclomethasone	8.Budesonide<br>9.Any other<br>__________	1.Heparin<br>2.LMWH<br>3.Warfarin<br>4.Apixaban	5.Rivaroxaban<br>6.Any other<br>___________	1.Deriphylline<br>2.Salbutamol<br>3.Salmeterol<br>4.Formoterol	5.Any other<br>_________

IX. Hematological and biochemical parameters *(Please enter first available value)**

**9.1.Complete blood count**		**9.2 Liver Function Tests**	
1.Hematocrit_____<br>2.Hb_____<br>3.TC_____	4.DC<br>N | L | M | E | B<br>5.ESR(30 mins)_____<br>6.Platelets_____	1.Albumin_____<br>2.Total protein____<br>3.AST(SGOT)_____<br>4.ALT(SGPT)_____	5.GGT_________<br>6.S. Bilirubin Direct ______<br>7.S. Bilirubin indirect______<br>8. S. Bilirubin Total_______

**9.3 Renal Function Test**		**9.4 Arterial blood gas values**	
1. Blood urea_________<br>2. Sr.Chloride_________<br>3.Sr.Creatinine_________<br>4.Sr.Sodium_________<br>5.Sr.Potassium_________	6.Sr. Uric acid_________<br>7.Sr. Calcium_________<br>8.BUN/Creatinine Ratio_____<br>9.Urea/Creatinine Ratio______	1. O2CT_________<br>2.Ph_________<br>3.PaCO2_________	4. PaO2_________<br>5.HCO3_________<br>6.O2Sat_________

**9.5 Blood sugar level**	**9.6 Acute phase reactants**		**9.7 Coagulation parameters**	
1.FBS_________<br>2.RBS _________<br>3.PPBS_________	1.CRP______<br>2.LDH______<br>3.D-Dimer_____	4.Ferritin_________<br>5.Transferrin_________<br>6.Procalcitonin_________	1.Bleeding time______<br>2.Clotting time_______<br>3.PT_________<br>4. INR_________	5.Aptt_________<br>6.Fibrinogen level______<br>7.Factor V assay_____

9.8. Sample for Blood culture collected	1. Yes, specify____ 2. No
9.9. Oral pharyngeal Swab –Bacterial culture	1. Yes, specify____ 2. No

X. Radiological investigation details*

10.1. X-Ray	1. Yes 2. No<br>Findings :<br>Severity score:
10.2. CT Scan	1. Yes 2. No<br>Findings :<br>Severity score:

XI. Treatment Outcome*

11.1. Final diagnosis	
11.2. Patients medical condition at discharge	1. Improved 2. Not improved
11.3.Final treatment outcome	1.Alive and discharged 2. Transferred to another facility 3. Palliative discharge 4. Death 5. LAMA
11.4.Date of discharge/ leaving from the hospital / death	dd/mm/yyyy
11.5.If admitted to ICU, the total duration of admission in ICU	___________days
11.6. If died, cause of death	1.Primary cause 2. Secondary cause(s) 3. Immediate cause 4. Antecedent cause<br>5. Other significant conditions(including comorbidities) contributing to death
11.7 If stable and discharged, continued medications:	
11.8. Outcome at one month after discharge	1.Alive and healthy 2. Alive with complications 3. Died 4. Unknown

XII. Virological testing*

12.1. Whether sample collected or not	1.Yes, sample ID____ 2. No
12.2. Date of sample collection	dd/mm/yyyy
12.2.1. Nasal swab	1. Yes 2. No
12.2.2. Throat swab	1. Yes 2. No
12.2.3. Nasopharyngeal swab	1. Yes 2. No

12.3 Viral Testing Result (Multiple)	1. Influenza A H1N1 2. Influenza A H1N1 with H275Y mutation 3. Influenza A H3N2 4. Influenza B-Yamagata 5. Influenza B-Victoria 6. SARS CoV-2 7. Any other_____________ 8. Negative

Name and signature of the Interviewer:___________________ Date:____________________

Verified By:___________________

CRF: Patients with Influenza Like Illness (ILI)

**CRF ID:**									

1.1. PHC / Hospital OPD No	
1.2. Date of enrolment	dd/mm/yyyy
1.3. Whether the patient has been interviewed for the same before?	1. Yes 2. No
1.4. Whether the patient satisfy ILI definition (an acute respiratory infection with measured fever >=38ºC or >=100.4ºFand cough with onset in the last 10 days)	1. Yes 2. No
1.5. Consent given: 1. Yes 2. No	1.6. Decision: Include 2. Exclude
**(Proceed further Only if Q1.6. is 1.Include)**	
1.7. Name	
1.8. Age (in completed years)	
1.9. Gender	1. Male 2. Female 3. Transgender
1.10. Contact Number of Patient	
1.11. Home Address	
1.12. Pin code	
1.13. Nature of locality	1. Rural 2. Urban 3. Slum
1.14. Height (cm)	
1.15.Weight (kg)	
1.16. Axillary temperature (C°/F°)	
1.17. MUAC (Under five) (cm):	
1.18. Currently Pregnant or not	1. Yes, gestational week(s)____________ 2. No
**II. Exposure History**	
2.1. Similar illness in family/neighbour/ workplace in last 14 days	1. Yes 2. No
2.2. Smoking (self)	1. Yes 2. No
2.3. Smoker in family	1. Yes 2. No
2.4. Any pets in the home	1. Yes, specify ______________________2. No
2.5. Exposure to birds / poultry/dead bird in last 14 days	1. Yes, specify _______________________2. No
2.6. Exposure to farm/live animal market/zoo/wild animals in the past 14 days	1. Yes, specify _______________________2. No
2.7. H/O attending any type of gatherings (wedding, parties, religious meetings etc. other than your workplace) in the past two weeks	1. Yes, specify _______________________2. No
2.8. In the past 14 days prior to symptoms onset, did you have any contact with a family member/relative/anyone in the neighbourhood or workplace having the similar illness	1. Yes, specify _______________________2. No
2.9. No. of persons living in the house______________	1. No. of children living in the house__________
2.10. H/o travel in past 14 days prior to onset of symptoms	1. Yes, specify _______________________2. No
2.11. H/o hospital / health facility visits for similar illness in the last 6 months	1. Yes, specify _______________________2. No
2.12. H/O lab confirmed COVID-19 positive in past one year	1. Yes 2. No
2.13. If COVID-19 positive, treatment availed	1. Home 2. In patient care
2.14. Cooking fuel used in the house	1. LPG 2. Firewood 3. Cow dung 4. Kerosene oil 5. Others___________________

2.15. Separate place(Kitchen) for cooking in the house	1. Yes 2. No		
2.16. Do you often undertake intercity/long-distance travel because of your job/family?	1. Yes 2. No		
2.17. If yes, the mode of travel used?	1. Personal vehicle 2 . Train 3. Bus 4. Air		
2.18.1. Influenza vaccination within last one year	1. Yes 2. No		
2.18.2. COVID-19 Vaccination	1. Yes 2. No		
If 'yes' type of vaccine received? 1. Covaxin, 2. Covishield, 3. Sputnik V, 4. Other, specify_________________ 5. Not Known			
		**If 'yes'-Name**	**If 'yes'-Date**
2.18.2.1. 1st dose	1.Yes 2. No		dd/mm/yyyy
2.18.2.2. 2nd dose	1.Yes 2. No		dd/mm/yyyy
2.18.2.3. Booster	1.Yes 2. No		dd/mm/yyyy
Routine immunization status (Under five)	1. Fully immunized 2. Partially immunized 3. Unimmunized 4. Not available		
2.19. First Symptoms(multiple)	1. Fever 2. Sore Throat 3. Cough 4. Cold 5. Diarrhoea 6. Others specify___________		
2.20. Date of onset of first symptoms	dd/mm/yyyy		
2.21. Rigors	1. Yes 2. No		
2.22. Sore throat	1. Yes 2. No		
2.23. Earache/ discharge	1. Yes 2. No		
2.24. Bodyache	1. Yes 2. No		
2.25. Chest Pain	1. Yes 2. No		
2.26. Vomiting/nausea	1. Yes 2. No		
2.27. Breathlessness/ difficulty breathing	1. Yes 2. No		
2.28. Seizures	1. Yes 2. No		
2.29. Chills	1. Yes 2. No		
2.30. Cough	1. Yes 2. No		
2.31. Haemoptysis	1. Yes 2. No		
2.32. Running nose	1. Yes 2. No		
2.33. Headache	1. Yes 2. No		
2.34. Malaise/Fatigue	1. Yes 2. No		
2.35. Abdominal pain	1. Yes 2. No		
2.36. Diarrhea	1. Yes 2. No		
2.37. Other symptoms, specify if yes __________	1. Yes 2. No		
2.38. Decreased feeding (under five)	1. Yes 2. No		
2.39. Lethargy/unconscious **(**Under five)	1. Yes 2. No		
**III. Past Medical History**			
3.1. Chronic lung disease	1. Yes 2. No		
3.2. COPD/Bronchitis	1. Yes 2. No		
3.3. Tuberculosis	1. Yes 2. No		
3.4. Diabetes	1. Yes 2. No		
3.5. Hypertension	1. Yes 2. No		
3.6. Chronic kidney disease	1. Yes 2. No		
3.7. Hematologic disorders	1. Yes 2. No		
3.8. Chronic diarrhoea in children under 5 year	1. Yes 2. No		
3.9. Asthma	1. Yes 2. No		
3.10. Cardiac Diseases	1. Yes 2. No		
3.11. Chronic liver disease	1. Yes 2. No		
3.12. Immunodeficiency disorders including HIV	1. Yes 2. No		
3.13. Chronic neurological disease including dementia	1. Yes 2. No		
3.14. Malignancy	1. Yes 2. No		
3.15. Other (specify)______________________	1. Yes 2. No		
**IV. Treatment Details**			
4.1. Have you taken any medicine after onset of ILI symptoms(before visiting PHC/Hospital)?	1.Yes,specify____ 2. No		
4.2. Antibiotics	1. Yes,specify____ 2. No		

	Starting date:dd/mm/yyyy	Weaning date:dd/mm/yyyy
4.3. Steroids	1.Yes,specify____ 2. No	
	Starting date:dd/mm/yyyy	Weaning date:dd/mm/yyyy
4.4. Bronchodilators	1.Yes,specify____ 2. No	
	Starting date:dd/mm/yyyy	Weaning date:dd/mm/yyyy
4.5. Outcome after 15 days	1.Cured2. Continuing Illness 3.Hospitalised 4. Died	
4.6. Date of resolution of symptoms (only for cured)	dd/mm/yyyy	
**V. Virological testing**		
5.1. Whether sample collected or not	1.Yes, sample ID____________________________________ 2. No	
5.2. Date of sample collection	dd/mm/yyyy	
5.3. Nasal swab	1. Yes 2. No	
5.4. Throat swab	1. Yes 2. No	
5.5. Nasopharyngeal swab	1. Yes 2. No	
5.6. Viral Testing Result	1.Influenza A H1N1 2. Influenza A H1N1 with H275Y mutation 3. Influenza A H3N2 4. Influenza B-Yamagata 5. Influenza B-Victoria 6. SARS CoV-2 7. Any other________ 8. Negative	

Name and signature of the Interviewer:______________________ Date:______________________

Verified by:____________________

Annexure 2

1.Facility checklist

PHC/ Block PHC/ Upgraded PHC/ Urban PHC/ District Hospital/ Government Headquarters Hospital/ Taluk Hospital/ Private hospital/ Private Medical College

1. General information

1.1 ID No:

1.2 District:

1.3 Block:

1.4 Facility name:

1.5 Facility type: PHC/ Block PHC/ Upgraded PHC/ Urban PHC/ District Hospital/ Government Headquarters Hospital/ Taluk Hospital/ Private hospital/ Private Medical College

1.6 Total Population catered by the facility: (skip for District Hospital, Private Hospital, Private Medical College)

1.7 Head of the institution/ Nodal person name:

1.8 Contact no:

2. Site description

2.1 Does the facility provide OPD services for ILI: Yes/ No

2.2 Does the facility provide IPD services for SARI: Yes/ No (skip for PHC/ Block PHC/ Upgraded PHC/ Urban PHC)

2.3 If **Yes** total no of beds available: (skip for PHC/ Block PHC/ Upgraded PHC/ Urban PHC)

2.4 If **Yes** Total beds earmarked for SARI out of total available beds: (skip for PHC/ Block PHC/ Upgraded PHC/ Urban PHC)

2.5 Are patients attending the facility from these groups: All age groups/ Only adults/ Only children

2.6 Average OPD for the last three months:

2.7 Average IPD for the last three months: (skip for PHC)

2.8 Does the facility currently report ILI cases: Yes/ No (Verify reports for last month) (If **No** exclude 2.9.1 and 2.9.2)

2.9.1 If **Yes** reporting through (portal): IDSP/ VRDLN/ IHIP/ Others (Please specify) (Verify reports for last month) (MCQ)

2.9.2 If **Yes** Reporting frequency: Daily/ Weekly/ Fortnightly/ Monthly/ Others (specify)

2.10 Does the facility currently report SARI cases: Yes/ No (Verify reports for last month) (skip for PHC/ Block PHC/ Upgraded PHC/ Urban PHC) (If **No for both 2.8 and 2.10** exclude 2.11- 2.16)

2.11 If **Yes** reporting through (portal): IDSP/ VRDLN/ IHIP/ Others (Please specify) (Verify reports for last month) (MCQ)

2.12 If **Yes** Reporting frequency: Daily/ Weekly/ Fortnightly/ Monthly/ Others (specify)

2.13 Whether standard case report forms are available exclusively for SARI/ ILI: Yes/ No

2.14 What key variables are reported: Name/ Age/ Gender/ Occupation/ Place/ Date of symptom onset/ Symptoms/ Date of consultation/ Date of sample collection/ Others (specify) (MCQ)

2.15 Any other method of reporting: WhatsApp /email/messenger/hard copies/phone/None (MCQ)

2.16 Immediate higher reporting unit: Block PHC/GH/District IDSP/Others (specify)

2.17 Does the facility has SOP/ Guidelines for infection prevention and control in place: Yes/ No

2.18 Does the facility have constituted an infection prevention and control committee (IPC)? Yes/ No

2.19 Does the facility conduct periodic IPC audits/ meetings: Yes/ No

2.20 Does the facility have established procedures and plans for emergency operations centre for outbreaks: Yes/ No

3. Human resources

3.1 Whether the facility has a dedicated person for compiling and sending reports: Yes/ No

3.2 If Yes, designation of the person responsible:

3.3 Personnel involved in ILI/ SARI surveillance in the facility

Personnel	Number	At least one person received formal training for ILI/ SARI surveillance (Y/ N)	Any refresher training on ILI/SARI received in the last year (Y/N)
Doctor			
Staff Nurse			
Lab technician			
DEO			
Health			

Inspector			
VHN, SHN, CHN			
Others (Specify)			

4. Infrastructure and method manuals

4.1 Does the facility have computers/ tablets: Yes/ No

4.2 Does the facility have internet connectivity: Yes/ No

4.3 Does the facility have SARI/ ILI registers: Yes/ No

4.4 Whether SOP/ Guidelines/ flowcharts for SARI/ ILI surveillance is available with the facility: Yes/ No

4.5 Whether Standard Treatment Guidelines for SARI/ ILI management is available with the facility: Yes/ No

4.6 Whether adequate facilities are available for treating ILI patients: Yes/ No

4.7 Whether adequate facilities are available for treating SARI patients: Yes/ No (skip for PHC/ Block PHC/ Upgraded PHC/ Urban PHC)

5. Outcomes

5.1 Does the facility receive any feedback from higher centres: Yes/ No

5.2 If **Yes,** type of feedback: Analysis reports/ Early Warning Signals/ Lab reports/ Outbreak notifications/ Quality assurance/ Performance reports (MCQ)

5.3 ILI cases treated at the facility in the last month: (Put not recorded if there is no data)

5.4 ILI cases reported from the facility in the last month: (Put not recorded if there is no data)

5.5 SARI cases treated at the facility in the last month: (Put not recorded if there is no data) (skip for PHC/ Block PHC/ Upgraded PHC/ Urban PHC)

5.6 SARI cases reported from the facility in the last month: (Put not recorded if there is no data) (skip for PHC/ Block PHC/ Upgraded PHC/ Urban PHC)

6. Lab:

6.1 Does the site have a laboratory: Yes/ No

6.2 If **No,** whether the facility has functional linkages with other laboratories: Yes/ No

6.3 Does the facility have a reliable power supply and fridge or a linked storage facility where the sample specimens can be kept: Yes/ No

6.4 Does the sample collector wear PPE during sample collection: No/ Only gloves/ medical mask and gloves/ only mask/ Full PPE/ Not available in the facility

6.5 Whether the facility collects samples for ILI surveillance: Yes/ No (If Yes, proceed further. For PHCs, etc. if Yes include 6.6, 6.9- 6.14. If No, go to 6.7. For PHCs, etc. stop)

6.6 Average number of ILI samples collected in the facility in last three months: (Put not recorded if there is no data)

6.7 Whether the facility collect samples for SARI surveillance: Yes/ No (skip for PHC/ Block PHC/ Upgraded PHC/ Urban PHC) (If Yes, proceed further, If No, stop)

6.8 Average number of SARI samples collected in the facility in last three months: (Put not recorded if there is no data) (skip for PHC/ Block PHC/ Upgraded PHC/ Urban PHC)

6.9 Samples collected out of reported cases: All/ some/ few/ none

6.10 Does your laboratory have facilities for testing ILI/SARI samples? Yes/No

6.11 If No, Frequency of sample transportation to testing lab? Daily/ Weekly/ Others (Specify)

6.12 If No, Mechanism of Sample transportation to the testing lab: Room temperature/ Ice packs/ Dry ice

6.13 Reporting through: IDSP/ VRDLN/ IHIP/ Others (Please specify) (Verify reports for last month) (MCQ)

6.14 Reporting frequency: Daily/ Weekly/ Fortnightly/ Monthly/ Others
(Specify)

6.15 Whether standard specimen collection forms are available exclusively for SARI/ ILI? Yes/ No

6.16 What key variables are reported with the specimen: Name/ Age/ Gender/ Occupation/ Place/ Date of symptom onset/ Symptoms/ Date of consultation/ Date of sample collection/ Type of sample/ Others (specify) (MCQ)

6.17 Any other method of reporting: WhatsApp /email/messenger/hard copies/phone/ None (MCQ)

2. Facility checklist for DPHL/ VDRLN/ MC lab/ DH lab / Private lab

Name of the laboratory		
Type of laboratory	DPHL/ VDRLN/ MCH lab/ DH lab/Pvt	
Is the lab is NABL accredited	Yes	No
Is the lab part of any ILI/ SARI/ ARI surveillance network?	IDSP/ IHIP/ ICMR/Other	
Whether testing clinical samples for Influenza and Covid-19?	1) Yes	2) No
What type of health facilities do you receive samples for testing?	PHC/ Block PHC/ CHC/ DH/MCH/ Private hospitals	
Total ILI/SARI samples tested in the last three month		
Functional computer available	1) Yes	2) No
Internet facility available	1) Yes	2) No
RT PCR available	1) Yes	2) No
If available no of RT-PCR		
Testing kits for Influenza and Covid-19 available	1) Yes	2) No
Laboratory biosafety level (BSL)		
Sample storage facility available?	1) Yes	2) No
Lab line list maintained (Register)	1) Yes	2) No
Consolidated data of samples available	1) Yes	2) No
Laboratory reports shared to DDHS / IDSP/ IHIP/ Other surveillance system	1) Yes	2) No
Laboratory reports shared to sample testing requesting facilities	1) Yes	2) No
Whether L form is maintained	1) Yes	2) No
L form reported through IDSP/IHIP	1) Yes	2) No
Whether the lab in enrolled in EQAS programmes	1) Yes	2) No
If **yes**, year of last EQAS done		
Whether the lab conducts regular IQAS checks?	1) Yes	2) No
Whether any lab personnel are involved in field level activities?	1) Yes	2) No

If **yes**, what type of activities	OBI/ Field training/ Routine monitoring

HR Abstract

Category	No of staff	Trained on SARI/ ILI Surveillance (Y/N)	If **yes,** type of training: Sample collection/ PCR training/ OBI/ Administrative/ Data analysis & reporting
Microbiologist			
Lab technician			
Lab assistant			
DEO			
Others			

3. Facility survey checklist for State Surveillance Unit/ District Surveillance Unit

1. General information

1.1 ID No:

1.2 Type of facility: SSU/ DSU

1.3 If DSU District name:

1.4 Nodal person name:

1.5 Contact no:

1.6 Date of survey:

2. Infrastructure

2.1 Whether dedicated space available: Yes/ No

2.2 Whether functional computers are available: Yes/ No

2.3 Whether internet connectivity is available: Yes/ No

2.4 Whether Guidelines/ SOPs for ILI/ SARI surveillance, reporting, analysis, and feed back is available: Yes/ No

2.5 How many EWS for ILI/ SARI have been picked up in the last one year :

2.6 How many outbreak responses have been conducted for ILI/SARI in the last one year:

3. Human resources

3.1 No of data entry operators:

3.2 No of data mangers:

3.3 No of epidemiologists:

3.4 Abstract of personnel involved in ILI/ SARI surveillance

Personnel	Number	Any formal training received on ILI/SARI surveillance
JD Epidemics		

DD Epidemics		
Epidemiologist		
Data manager		
DEO		
Others (Specify)		

5. Processes and outcomes

5.1 Total number of reporting units:

5.2 No of reporting units reported ILI/SARI in the last three months:

5.3 No of reporting units with complete reports in IDSP/IHIP in the last three months:

5.4 Abstract of reporting units

Reporting units	Numbers	AFI reporting units	ILI reporting units	SARI reporting units	Units having DEO	No of supervisory visits conducted in last 3 months
Govt MCH						
Private MCH						
ESI hospitals						
Block PHC						
Government Hospitals						
District Hospitals						
PHC						
Additional PHC						

5.5 Abstract of data analysis

**In the past 3 months**	**Daily (Y/N)**	**Weekly (Y/N)**	**Fortnightly (Y/N)**	**Monthly (Y/N)**	**Not done (Y/N)**
ILI analysis done for reporting					
ILI analysis done for time/place/person					
AFI analysis done for reporting					
AFI analysis done for time/place/person					
SARI analysis done for reporting					
SARI analysis done for time/place/person					

5.6 Feedback given to reporting units : Yes/ No (verify reports for last 3 months)

5.7 If Yes what type of feedback: Analysis reports/ EWS/ Outbreak notifications/ Quality assurance/ Performance reports.

Establishing a model for integrated Influenza surveillance in Tamil Nadu, India

4. Interview Guide for District Epidemiologist/ Data manager/ Statistical officer (Epidemiology)/ Hospital Superintendent

Participant ID: Date:
Designation:
Name of the institution/ health centre:
Place:
District:
Duration of service: ________ years
Start time:
End time:

1. Do you think that there is a need for SARI/ ILI surveillance system in the district?
   [Probe: uses of influenza surveillance, containing the seasonal outbreaks, policy changes, flu experience, Covid experience, EWS, competing interests (MCH, NCD, other CDs) etc.]

2. Is there an existing surveillance system for SARI/ ILI? If yes, could you please elaborate on the existing SARI/ILI surveillance system in the district?
   [Probe: data collection, quality and reporting, training, frequency of report, IHIP portal, monitoring, feedback, funding source, etc.]

3. Who are the other stakeholders/other departments involved in the SARI/ILI surveillance?
   [Central agency, Directorates, NCDC, ICMR, Public welfare department, Police and fire, water board, animal husbandry, media, environment, forest, education, NGOs, private, challenges in the interdepartmental coordination, data sharing across departments, etc.]

4. Could you please share your experiences in SARI/ILI surveillance?
   [Probe: data collection, compilation, entry, reporting, quality of data, validation of data analysis and feedback, challenges, guidelines, supervision]

5. Can you elaborate upon the surveillance trainings that you may have received?
   (Probe: Challenges in the training, frequency, IEC, SOP/ guidelines, outbreak investigation, etc.)

6. Could you please tell us about the response activities undertaken based on the SARI/ILI surveillance data? *(only for DE)*
   [Probe: District and state level, based on the outbreaks, facility, local, state level, awareness, infrastructure development, immunization, treatment etc.]

7. Could you please tell us the challenges in establishing/ implementing the SARI/ILI surveillance in the district? *(only for DE)*
   [Probe: HR, geography, infrastructure, laboratory infrastructure, community involvement, IEC, portals, quality of data, analysis and response activities, challenges in data collection, data entry, training, funding issues]

8. Could you please give your suggestions/inputs to improve/establish the SARI/ILI surveillance in the district?

5. Interview Guide for DPHPM/ JD (Epidemics)/HOD/Dean/DDHS

Participant ID:
Date:
Designation:
Name of the institution/ health centre:
Place:
District:
Duration of service: ________ years
Start time:
End time:

1. Do you think that there is a need for SARI/ ILI surveillance system in the state?
   [Probe: uses of influenza surveillance, containing the seasonal outbreaks, policy changes, flu experience, Covid experience, EWS, competing interests (MCH, NCD, other CDs), etc.]

2. Is there an existing surveillance system for SARI/ ILI? If yes, could you please elaborate on the existing SARI/ILI surveillance system in the state?
   [Probe: data collection, quality and reporting, training, frequency of report, IHIP portal, monitoring, feedback, funding source, etc.]

3. Who are the other stakeholders/other departments involved in the SARI/ILI surveillance?
   [Central agency, Directorates, NCDC, ICMR, Public welfare department, Police and fire, water board, animal husbandry, media, environment, forest, education, NGOs, private, challenges in the interdepartmental coordination, data sharing across departments, etc.]

4. What response activities have been undertaken based on the SARI/ILI surveillance data in the state?
   [Probe: based on the outbreaks, facility, local, state level, awareness, infrastructure development, immunization, treatment etc.]

5. Could you please tell us the challenges in establishing the SARI/ILI surveillance in Tamil Nadu?
   [Probe: HR, geography, infrastructure, laboratory infrastructure, community involvement, IEC, portals, quality of data, analysis and response activities, challenges in data collection, data entry, training, funding issues]

6. Could you please give your suggestions/inputs to establish/ improve the SARI/ILI surveillance in the state?

6. Interview Guide for Microbiologist - Laboratory (DPHL/ VRDL/ Medical College Lab/ Private Hospital Lab)

Participant ID:
Date:
Designation:
Name of the institution/ health centre:
Place:
District:
Duration of service: ________ years
Start time:
End time:

1. Do you think that there is a need for SARI/ ILI surveillance system in the district?
   [Probe: uses of influenza surveillance, contain the seasonal outbreaks, policy changes, flu experience, Covid experience, EWS, competing interests (MCH, NCD, other CDs) etc.]

2. Is there an existing surveillance system for SARI/ ILI? If yes, could you please elaborate on the existing SARI/ILI surveillance system in the district?
   [Probe: data collection, quality and reporting, training, frequency of report, IHIP portal, monitoring, feedback, funding source, etc.]

3. Could you please share your experience in SARI/ILI surveillance?
   [Probe: data collection, compilation, entry, reporting, quality of data, validation of data analysis and feedback, challenges, guidelines, supervision]

4. Could you please tell us the challenges in establishing/ operating SARI/ ILI laboratory surveillance?
   [Probe: HR, infrastructure, consumables, training, budget, procurement, quality assurance, sample collection, storage, processing, result analysis, reports, feedback, collaboration/ co-operation/ co-ordination]

5. Could you please give your suggestions/inputs to improve/establish the SARI/ILI surveillance in the district?

6. Are you testing SARI/ ILI samples (Influenza, Covid-19) and notifying the positive results to the state health authorities? (Probe: challenges in sharing results, patient confidentiality, burden of work, costs involved)

Establishing a model for integrated Influenza surveillance in Tamil Nadu, India

7. Questionnaire for Data manager/ Data Analyst [Private Labs]

Participant ID: Date:

No.	Questions	Responses
	**Facility information**	
1.	Name of the District	________________
2.	Name of Block	________________
3.	Place of facility	________________
4.	Type of the facility	1) PHC<br>2) Upgraded PHC<br>3) Urban PHC<br>4) Block PHC<br>5) GH/Taluk hospital<br>6) District hospital<br>7) Corporation hospital<br>8) Private Hospital<br>9) Medical college<br>10) Others
	**Socio-demographic information**	
5.	Age (In completed years)	
6.	Gender	1) Male<br>2) Female<br>3) Others
7.	Highest education	1) Diploma<br>2) Degree<br>3) Post graduate<br>4) Ph.D.<br>5) Others_____
8.	Designation	______________
9.	Total years of experience<br>(in years)	______________
10.	Total years of experience in this facility<br>(in years)	______________

	**SARI/ILI surveillance**	
11.	Did you receive any SARI/ ILI data management and analysis training in the last 2 years?	1) Yes<br>2) No
12.	Are you familiar with IDSP/ IHIP portal for SARI/ ILI reporting?	1) Yes<br>2) No<br>3) Not sure
13.	Are you familiar with any other reporting surveillance system for SARI/ILI data?	1) Yes<br>2) No<br>3) Not sure
14.	If yes, please mention	______________
15.	Are you performing any data analysis in your facility based on the SARI/ILI cases?	1) Yes<br>2) No
16.	If yes, what are the key characterises of the analysis report?	1) Incidence<br>2) Prevalence<br>3) Death count<br>4) Time place and person analysis<br>5) Reporting of line list<br>6) EWS<br>7) Others_________
17.	If yes, how frequently are you performing data analysis and reporting (consolidated analysis report)?	1) Daily<br>2) Weekly<br>3) Monthly<br>4) Others_________
18.	Are you generating any EWS/ cluster/ outbreak reports?	1) Yes<br>2) No<br>3) Not sure
19.	Do you get any feedback from higher officials based on the SARI/ILI surveillance report?	1) Yes<br>2) No<br>3) Not sure
20.	If yes, what type of feedback do you receive? *(Multiple choice)*	1) Early warning signals<br>2) Clusters<br>3) Outbreaks<br>4) Performance<br>5) Others ________
21.	Do you provide any feedback to reporting units (Block and PHC level) based on received SARI/ILI data?	1) Yes<br>2) No<br>3) Not sure

22.	If yes, what type of feedback do you provide? *(Multiple choice)*	1) Early warning signals<br>2) Clusters<br>3) Outbreaks<br>4) Performance<br>5) Others ________

8. Questionnaire for Lab technician

Participant ID: Date:

No.	Questions	Responses
	**Facility information**	
1.	Name of the District	_________________
2.	Name of Block	_________________
3.	Place of facility	_________________
4.	Type of the facility	11) PHC<br>12) Upgraded PHC<br>13) Urban PHC<br>14) Block PHC<br>15) GH/Taluk hospital<br>16) District hospital<br>17) Corporation hospital<br>18) Private Hospital<br>19) Medical college<br>20) Others_____________
	**Socio-demographic information**	
5.	Age (In completed years)	
6.	Gender	4) Male<br>5) Female<br>6) Others

7.	Highest education	1) Diploma<br>2) Degree<br>3) Post graduate<br>4) Ph.D<br>5) Others_____
8.	Total years of experience<br>(in years)	______________
9.	Total years of experience in this facility<br>(in years)	______________
	**SARI/ILI surveillance**	
10.	Are you aware of SARI/ ILI case definitions?	1) Yes<br>2) No
11.	Do you think SARI/ ILI surveillance is important?	1) Yes<br>2) No<br>3) Don’t know
12.	Have you attended any SARI/ILI surveillance trainings in last two years?	1) Yes<br>2) No
13.	Have you attended any other surveillance trainings in last two years?	1) Yes<br>2) No
14.	Do you maintain a lab line list register	1) Yes<br>2) No
15.	Do you maintain a register for samples collected	1) Yes<br>2) No
16.	Are you familiar with IDSP/ IHIP portal?	1) Yes<br>2) No
17.	Do you report SARI/ILI cases using L form?	1) Yes<br>2) No
18.	Do you report SARI/ILI cases in any other portal?	1) Yes<br>2) No
19.	If yes please mention	

9. Questionnaire for MO/ BMO/ Specialists/ IDSP nodal persons

Participant ID: Date:

No.	Questions	Responses
	**Facility information**	
20.	Name of the District	_________________
21.	Name of Block	_________________
22.	Place of facility	_________________
23.	Type of the facility	21) PHC<br>22) Upgraded PHC<br>23) Urban PHC<br>24) Block PHC<br>25) GH/Taluk hospital<br>26) District hospital<br>27) Corporation hospital<br>28) Private Hospital<br>29) Medical college<br>30) Others
	**Socio-demographic information**	
24.	Age (In completed years)	
25.	Gender	7) Male<br>8) Female<br>9) Others
26.	Highest education	6) MBBS<br>7) Diploma (Specialization)<br>8) MD<br>9) MPH<br>10) Ph.D.<br>11) Others_____
27.	Designation	_______________
28.	Total years of experience (in years)	_______________

29.	Total years of experience in this facility<br>(in years)	______________
	**SARI/ILI surveillance**	
30.	Are you aware of SARI/ ILI case definitions?	3) Yes<br>4) No
31.	How often do you encounter SARI/ ILI cases in your facility?	1) Never<br>2) Daily<br>3) Weekly<br>4) Occasionally<br>5) Others_____
32.	Do you think SARI/ ILI cases should be reported to concerned authorities?	1) Yes<br>2) No<br>3) Don't know
33.	Do you think SARI/ ILI surveillance is important?	4) Yes<br>5) No<br>6) Don't know
34.	Did you receive any training related to SARI/ ILI surveillance in the last 2 years?	1) Yes<br>2) No
35.	When did you last receive training on SARI/ ILI surveillance?	________________
36.	How many SARI/ ILI training programs have you attended?	________________
37.	Have you attended any other surveillance trainings in last two years?	3) Yes<br>4) No
38.	Are you familiar with IDSP/ IHIP portal for SARI/ILI reporting?	3) Yes<br>4) No<br>1) Not sure
39.	Are you aware of any other reporting system for SARI/ ILI?	1) Yes<br>2) No
40.	If yes, please mention	________________
41.	Did you attend any review meetings regarding SARI/ ILI surveillance in the last six months?	1) Yes<br>2) No
42.	Could you please explain your experience in implementing SARI/ILI surveillance and challenges that you are facing.	
43.	To improve ILI/SARI surveillance, what are your needs/ requirements?	

10. Questionnaire for VHN/UHN/SHN/HI/ASHA/Nurse

Participant ID: Date:

No.	Questions	Responses
	**Facility information**	
1.	Name of the District	________________
2.	Name of Block	________________
3.	Place of facility	________________
4.	Type of the facility	31) HSC<br>32) PHC<br>33) Upgraded PHC<br>34) Urban PHC<br>35) Block PHC<br>36) GH/Taluk hospital<br>37) District hospital<br>38) Corporation hospital<br>39) Private Hospital<br>40) Medical college<br>41) Others
	**Socio-demographic information**	
5.	Age (In completed years)	
6.	Gender	10) Male<br>11) Female<br>12) Others
7.	Highest education	1) Diploma<br>2) Degree<br>3) Post graduate<br>4) Ph.D<br>5) Others_____
8.	Designation	______________
9.	Total years of experience<br>(in years)	______________
10.	Total years of experience in this facility<br>(in years)	______________

	**SARI/ILI surveillance**	
11.	Are you aware of SARI/ ILI cases definitions?	1) Yes<br>2) No
12.	How often have you encountered SARI/ ILI cases in your facility?	1) Never<br>2) Daily<br>3) Weekly<br>4) Occasionally<br>5) Others_____
13.	Do you think SARI/ ILI cases should be reported to higher authorities?	1) Yes<br>2) No<br>3) Don't know
14.	Do you **refer** SARI/ ILI cases to the higher facility from the community/ field? *(for VHN/ HI)*	1) Yes<br>2) No<br>3) Don't know
